# Spatial analysis of intracerebral electroencephalographic signals in the time and frequency domain: identification of epileptogenic networks in partial epilepsy

**DOI:** 10.1098/rsta.2008.0220

**Published:** 2008-10-28

**Authors:** Fabrice Wendling, Fabrice Bartolomei, Lotfi Senhadji

**Affiliations:** 1 INSERMU642, Rennes 35000, France; 2 Université de Rennes 1, LTSIRennes 35000, France; 3 INSERM, U751Marseille 13000, France; 4 Faculté de Médecine, Aix Marseille UniversitéMarseille 13000, France; 5 Service de Neurophysiologie Clinique, AP-HM, Hôpital de la TimoneMarseille 13000, France

**Keywords:** electroencephalography, intracerebral, epilepsy, interictal, ictal, statistical signal processing

## Abstract

Electroencephalography (EEG) occupies an important place for studying human brain activity in general, and epileptic processes in particular, with appropriate time resolution. Scalp EEG or intracerebral EEG signals recorded in patients with drug-resistant partial epilepsy convey important information about epileptogenic networks that must be localized and understood prior to subsequent therapeutic procedures. However, this information, often subtle, is ‘hidden’ in the signals. It is precisely the role of signal processing to extract this information and to put it into a ‘coherent and interpretable picture’ that can participate in the therapeutic strategy. Nowadays, the panel of available methods is very wide depending on the objectives such as, for instance, the detection of transient epileptiform events, the detection and/or prediction of seizures, the recognition and/or the classification of EEG patterns, the localization of epileptic neuronal sources, the characterization of neural synchrony, the determination of functional connectivity, among others. The intent of this paper is to focus on a specific category of methods providing relevant information about epileptogenic networks from the analysis of spatial properties of EEG signals in the time and frequency domain. These methods apply to either interictal or ictal recordings and share the common objective of localizing the subsets of brain structures involved in both types of paroxysmal activity. Most of these methods were developed by our group and are routinely used during pre-surgical evaluation. Examples are detailed. Results, as well as limitations of the methods, are also discussed.

## 1. Introduction

Epilepsy is a neurological disease that directly affects 50 million people worldwide (Prilipko *et al*. 2006). It is characterized by the recurrence of seizures that markedly reduce the patient's quality of life. Epilepsy is a complex disease because there are many possible causes for seizures. In fact, any disturbance of the normal neuronal activity due to illness, brain damage or abnormal brain development can provoke seizures and subsequent epilepsy (Hauser & Lee 2002).

In 20–30 per cent of the cases, anti-epileptic drugs do not allow for efficient control of seizures. In most patients, these drug-resistant epilepsies are ‘partial’ or ‘focal’, i.e. seizures are generated in a hyperexcitable brain region, often referred to as the epileptogenic zone (EZ), located in one or both hemispheres. In partial epilepsies, the localization and the precise definition of the EZ are the two main issues to be solved in order to propose to the patient an alternative therapeutic strategy such as surgery which, for these patients, may at present be the only option to suppress seizures. Therefore, during the pre-surgical evaluation of patients with drug-resistant partial epilepsy, a number of clinical investigations providing both anatomical (presence of a lesion, for instance) and functional (presence of abnormalities in brain electrical or metabolic activity, for instance), data are performed in order to determine the organization of the EZ, which often corresponds to a network of structurally and functionally connected brain structures (Bragin *et al*. 2000; Bartolomei *et al*. 2001; Briellmann *et al*. 2004). Among these investigations, electroencephalography (EEG; scalp EEG) and stereoelectroencephalography (SEEG; intracerebral EEG) consist in the measurement of brain electrical activity using electrodes positioned either on the head or directly implanted into brain structures, respectively, potentially involved into the generation of epileptic events, during either ictal periods (seizures) or interictal periods (outside seizures). Therefore, in a given patient, EEG signals contain essential information about the topology of his/her epileptogenic network. This information must be ‘extracted’ from signals and must be ‘decoded’ by clinicians in order to define the best surgical procedure aimed at suppressing seizures while keeping the patient's cognitive, sensory and/or motor functions intact.

The visual inspection of intracerebral EEG signals remains a difficult task, especially in the context of pre-surgical evaluation where epileptic patients are monitored for several days, 24 hours a day. This visual inspection can be complemented by the use of signal analysis methods. Indeed, such methods can facilitate the processing of large amounts. They can also quantify some signal features that can hardly be obtained visually (such as the coherence between signals recorded from distant sites). Although the concept of ‘quantified EEG’ started in the early 1960s, the past decades have seen a considerable development of digital signal processing methods due, in particular, to the use in clinical practice of digitized video–EEG monitoring systems that have progressively replaced analogous systems since the 1990s. Nowadays, the panel of available methods is very wide depending on the objectives such as, for instance, the detection of transient epileptiform events (Senhadji & Wendling 2002), the detection and/or prediction of seizures (Gotman 1999; Lehnertz *et al*. 1999; Iasemidis 2003), the recognition and/or the classification of EEG patterns (Creutzfeldt *et al*. 1985; Wendling *et al*. 1997), the localization of epileptic neuronal sources (Ebersole & Hawes-Ebersole 2007), the characterization of neural synchrony (Uhlhaas & Singer 2006), the determination of functional connectivity (Stam *et al*. 2007), among others.

The intent of this paper is not to cover all the aforementioned topics but to concentrate on a specific category of methods providing relevant information about the EZ from the analysis of time–frequency and spatial properties of EEG signals (here, the terminology ‘spatial’ refers to the concept of interdependency between signals recorded from distinct brain regions). Some of the methods presented in this paper can be applied to transient events (epileptic spikes) occurring during interictal periods, some can be applied to the ongoing EEG activity, in particular during the transition from interictal to ictal activity. In all cases, as used in the context of epilepsy, the general purpose of reviewed methods is to derive relevant information about the topology of neural networks involved in both types of paroxysmal activity from invasive (intracerebral EEG) observations. Detailed examples are based on methods that are routinely used during pre-surgical evaluation. These methods provide interpretable and useful information about epileptogenic networks and therefore about the subsequent surgical procedure to be performed in the singularity of the problem posed by each patient.

To end with, it is noteworthy that some other approaches that are also used during pre-surgical evaluation for focus localization and extent are not dealt with in this paper as spatial methods developed in the field of epilepsy over the past decades (see Michel *et al*. 2004 for review). Along these lines, we did not report results about linear methods (based on multivariate autoregressive model) proposed to estimate causality (in the Granger sense) between signals such as the directed coherence method or the partial directed coherence, among others (see Gourevitch *et al*. 2006 for review on potential applications in neurophysiology).

## 2. Intracerebral EEG recording in partial epilepsies

Generally speaking, EEG is used for both clinical and research purposes. It consists in measuring the electrical activity of the brain using electrodes positioned on the surface of the head (i.e. scalp). From biophysical considerations, it is known that the EEG is mainly sensitive to the post-synaptic activity of neuronal cells aligned in space, such as the pyramidal cells of the neocortex (organized in ‘palisades’), for instance (Lopes da Silva 2002). In partial epilepsies, for pre-surgical evaluation purposes, direct recording from intracerebral electrodes can also be performed.

**Figure 1:**
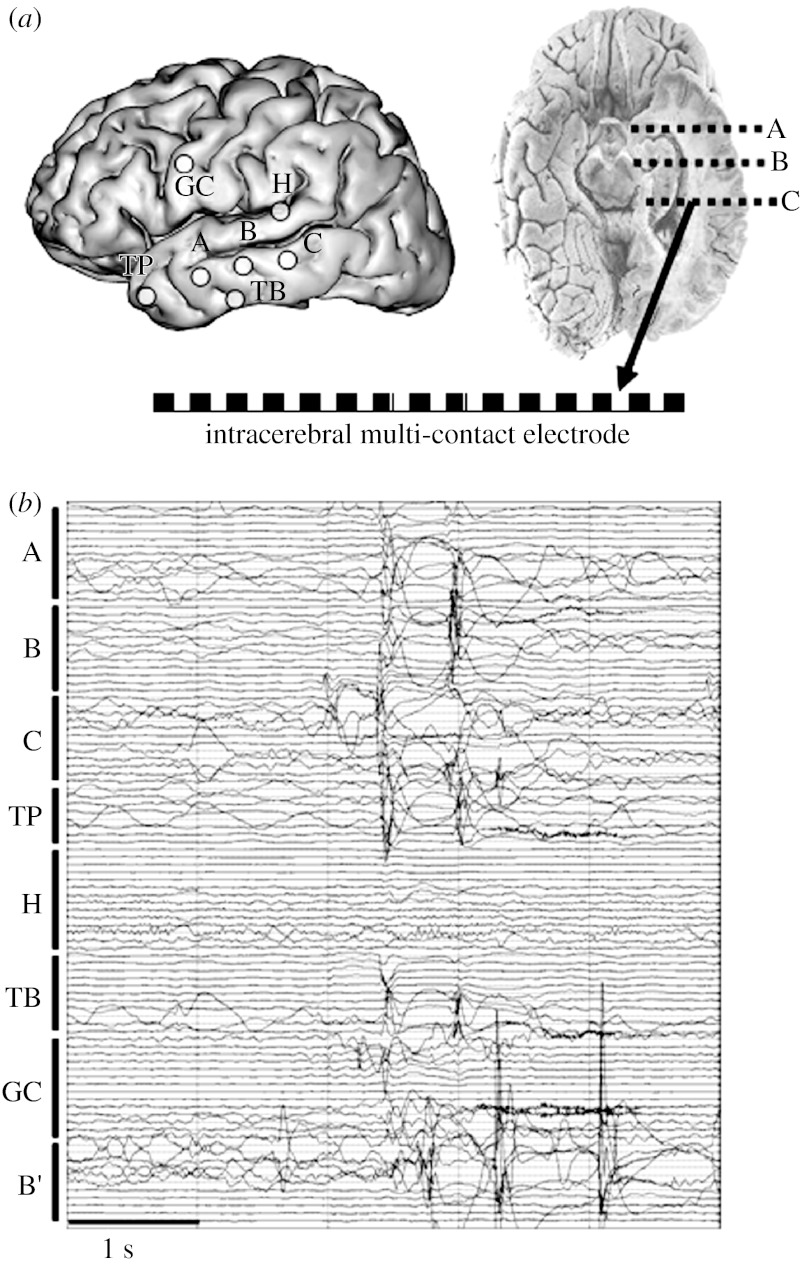
(*a*) SEEG recording technique used during pre-surgical evaluation of patients with drug-resistant epilepsy. (*b*) An example of intracerebral EEG recording performed in a patient with TLE (interictal activity). Capital letters A, B, C, …, B′ refer to electrode labels. Targeted structures: A, amygdala and middle temporal gyrus; B, anterior hippocampus and middle temporal gyrus; C, posterior hippocampus and middle temporal gyrus; TP, temporal pole; H, insula and superior temporal gyrus; TB, temporo-basal cortex; GC, cingular gyrus; B′, anterior hippocampus (contra-lateral electrode).

In the context of partial epilepsies, most of the clinical units make routine use of 23–32 scalp electrodes. The sampling rate of EEG signals also dramatically increased during the past years as the presence of fast (gamma frequency band, 30–80 Hz) and very fast activity (beyond gamma) might be a signature of some particular types of epilepsy (Rampp & Stefan 2006). The analysis of scalp EEG recordings (always performed in conjunction with the video) is intended to extract information on the presence of epileptiform events in interictal periods (epileptic spikes, spike waves), on the presence of abnormal rhythms (slow waves), on lateralization (i.e. the hemisphere in which seizures start), on the approximate localization of the EZ in a given brain region (frontal, temporal). Such information provides essential arguments to determine which brain structures are potentially involved in the epileptogenic network. It is thus directly used to define the position of intracerebral electrodes prior to surgery. Indeed, intracerebral EEG recording may be necessary when hypotheses about the precise location and organization of the EZ (formulated from non-invasive data) are not sufficient to define the surgical procedure (brain resection). Several techniques are available for direct recording of the brain activity depending on the type of electrode that is used and on the way electrodes are positioned in target brain structures. One of the gold standards is the ‘SEEG’ introduced by Bancaud and Talairach in the 1960s (Bancaud & Talairach 1973). This technique is illustrated in figure 1. It is based on the stereotaxic registering of the target anatomical structures and allows for recording of electrical activity within the intracranial space. This attempt to characterize the epileptic activity according to a three-dimensional topography remains in contrast to the other existing neurophysiological methods of pre-surgical exploration based on electrocorticography (ECoG) that uses subdural grid electrodes. The SEEG has been therefore developed in part with the aim of overcoming certain limitations linked with the two-dimensional and surface features of ECoG recording. The core of the method is the ‘anatomo-electro-clinical correlation’: a close analysis of clinical signs (semiology) and their relationship with the regions primarily and secondarily involved in the epileptic discharge. The aim is to define a temporo-spatial profile of the seizure's origin and propagation, thus aiding the decision for accurate surgical intervention to be performed.

## 3. From intracerebral EEG signals to epileptogenic networks

### (a) Identification of subsets of structures involved in interictal activity

#### (i) Research context and problem statement

As mentioned previously, two types of epileptic activity are reflected in EEG signals: seizures and paroxysmal transient events, often referred to as ‘interictal spikes’ when they show a sharp component. The exact relationship between networks of structures involved in both types of activity is a recurrent question in epileptology. Although this relationship remains unclear, the analysis of interictal events is complementary to the analysis of seizures and is recognized as useful in the study of the EZ. For the past decades, numerous studies have been performed on interictal paroxysmal events both in human (Talairach & Bancaud 1966) and animal models (Avoli & Barbarosie 1999). Their morphology was first studied by Penfield & Jasper (1954), who introduced two classes (primary and propagated) and found that sharper spikes were markers of the epileptogenic lesion. A central question was raised about the characterization of their spatio-temporal distribution, both in surface and in intracerebral EEG signals (Barth *et al*. 1984; Chauvel *et al*. 1987; Stefan *et al*. 1990). Mapping techniques were developed (Badier & Chauvel 1995) and revealed the origin of interictal spikes as well as possible propagation schemes. Several studies based on intracerebral recording in humans also suggested that regions leading interictal activity may match seizure onset zones (Hufnagel *et al*. 2000; Asano *et al*. 2003). Their identification could therefore help to tailor resections in order to improve seizure control, as suggested earlier in Alarcon *et al*. (1997).

This brief literature review indicates that the co-occurrence of interictal spikes generated within distant structures can be considered key information. It also shows that questions related to the organization of transient epileptic events (in time and space) remain open: in a given patient, what are the cerebral structures involved during these events? Are these structures involved in a reproducible way? How can reproducibility be objectively characterized? The central question to be solved from multichannel depth EEG signals is therefore to automatically identify the multiple structures that are conjointly involved in the generation of transient epileptic events, in a reproducible way. This problem is particularly complex if one considers (i) the tremendous amount of spikes (up to several thousands per hour) that can be recorded during pre-surgical evaluation which usually lasts for 5 to 8 days and (ii) the apparent variability of electrophysiological patterns (in terms of EEG waveforms and involved brain structures during interictal events).

#### (ii) Method: automatic identification of subsets of co-activated structures

**Figure 2:**
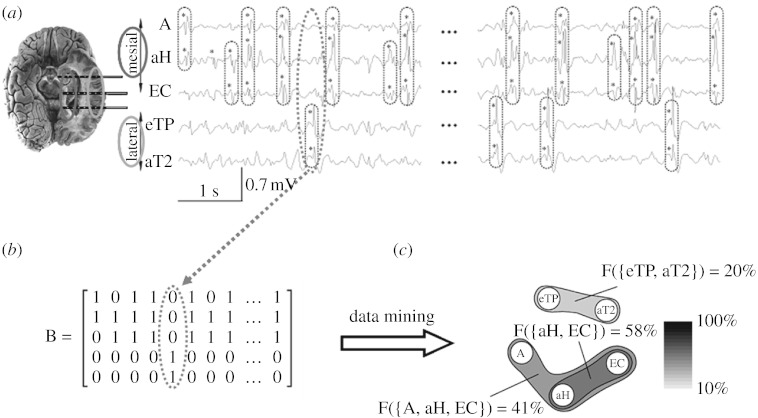
(*a*) Detection of mono-IIS and identification of multi-IIS in intracerebral EEG, (*b*) Boolean co-occurrence matrix and (*c*) extraction and representation of SCAS. Signal processing method for identifying subsets of structures involved in interictal epileptic events (adapted from Bourien *et al*. 2005). See text for details.

Only few information processing methods able to statistically characterize the spatio-temporal distribution of interictal transient events in large datasets have been reported up to now. The objective of this section is to present the approach proposed in Bourien *et al*. (2004, 2005) that can be seen as a complement to the visual analysis performed by the epileptologist. From the processing of long-duration intracerebral EEG recordings, this approach can automatically extract the subsets of brain structures frequently and conjointly involved in the generation of intracerebral interictal spikes (referred to as ‘subsets of co-activated structures’ or ‘SCAS’). The approach is summarized in figure 2. It proceeds according to the three steps that are briefly described below: (i) the automatic detection of monochannel intracerebral interictal spikes (mono-IIS), (ii) the formation of multichannel intracerebral interictal spikes (multi-IIS), and (iii) the automatic extraction of SCAS that makes use of a data-mining algorithm and statistical tests.

##### Detection of mono-IIS

Mono-IIS are transient events frequently observed in EEG signals recorded in epileptic patients. The detection of interictal spikes is considered to be a difficult problem. It has been, and is still, the topic of a large number of publications in the field of EEG analysis. Many algorithms have been proposed based on Fourier or wavelet transforms, on mimetic and rule-based approaches, on neural networks, on adaptive filtering (template matching), on principal or independent component analysis. Readers may refer to Gotman (1999), Senhadji & Wendling (2002) and Tzallas *et al*. (2006) for partial reviews. As shown in many studies, even very recent ones (Brown *et al*. 2007), the ideal spike detector does not exist, as the specificity and the sensitivity remain difficult to control in a context where (i) the frequency of spikes is modulated by the patient state (Gotman & Wang 1992) and (ii) where human experts have themselves difficulties, in some cases, to assess the presence of spikes in EEG signals (Dumpelmann & Elger 1999).

From the electrographic viewpoint, interictal events are generally characterized by a sharp component called a spike, sometimes followed by a slow wave (Chatrian *et al*. 1974). The spike component (the ‘useful signal’) is high amplitude and short duration compared with background EEG activity (the ‘noise’). In this section, we present a time–frequency domain method that makes use of a first stage for enhancing the signal-to-noise ratio and of a second stage for deciding whether or not a spike is present. The first stage is based on a wavelet decomposition that has proven particularly suited for enhancing transient signals well localized in time and frequency. More specifically, a quadratic approach is used in which the cumulative value *q*(*t*) of squared modulus of outputs of a wavelet filter bank is computed at each sample time *t* (Senhadji *et al*. 1995). The amplitude of quantity *q*(*t*) is random and depends on the signal content. During background EEG, its mean value is low. Conversely, during spike episodes, its mean value becomes higher as coefficients associated with high-frequency bands transiently increase. This behaviour is used in the second stage, which makes use of a Page–Hinkley change-point algorithm (Basseville & Nikiforov 1993) in order to automatically estimate time instants corresponding to abrupt changes of *q*(*t*). In this method, two parameters (the bias and the threshold) are to be adjusted separately to adapt detection performances (in terms of false negatives and false positives). Using simulations (not reported here), the authors showed that this detection method produces only few false negatives even if the rate of false positives is higher than other methods. This feature was considered as acceptable since it minimizes the lost information (spikes not detected) and consequently has a limited effect on the next steps of the entire procedure of SCAS extraction. For the first stage of the whole detection procedure, the authors also found that performances obtained for a wavelet-based approach were similar to those obtained for a Gabor decomposition-based approach in which filters have a uniform bandwidth (see for example Senhadji *et al*. (1997) in which different quadratic spike detectors are quantitatively compared).

##### Identification of multi-IIS

The purpose of this step is to identify multi-IIS, defined as electrographic events appearing in multichannel EEG signals and including at least two mono-IIS that ‘co-occur’ in the same temporal interval of duration *D*. Typically, this issue can be solved using an algorithm based on a window of duration *D* sliding on multichannel EEG signals, after detection of mono-IIS (previous step), as described by Bourien *et al*. (2004). This algorithm leads to the construction of a Boolean matrix whose element is equal to 1 if a mono-IIS is present on channel within the *j*th multi-IIS, and is equal to zero otherwise. The columns of matrix *B* contain the co-occurrence information extracted from multichannel EEG data. This information is the input of the third step on the procedure aimed at extracting SCAS. In the following, this matrix is referred to as the ‘co-occurrence Boolean matrix’. In the identification of multi-IIS, the critical parameter is *D*, which is dependent on both the duration of multi-IIS and the time separating distinct multi-IIS. It must be sufficiently short to avoid fusion of temporally unrelated monochannel events but it must be long enough to avoid the situation where temporally related events are not detected. Experimentally, the authors obtained good extraction of multi-IIS in mesial temporal lobe epilepsy for *D* values ranging between 100 and 250 ms in accordance with range values indicated in Alarcon *et al*. (1994). *D* was then chosen to be equal to 150 ms.

##### Extraction of SCAS

This third step consists in extracting, from matrix *B*, ‘maximal’ and ‘frequent’ SCAS, i.e. the subsets including a maximal number of distant brain structures that frequently co-activate during transient events. A straightforward exhaustive method for solving this problem is to classify all subsets present in *B* (each class corresponding to a set of column vectors with the same coordinates equal to 1) and to compute the frequency of occurrence of each class. However, for a high number *N* of channels, the algorithmic complexity becomes very high. As described in Bourien *et al*. (2004), an alternative solution is to use algorithmic techniques coming from data mining that were proposed to efficiently extract frequent sets of items (or ‘itemsets’) from large databases with reduced computation time. Such techniques were initially developed by Agrawal & Srikant (1994) and Mannila *et al*. (1994) who first proposed iterative methods that considerably reduce the number of candidate itemsets to be tested at each step. The key idea starts from the fact that (i) a set of *n* items (or ‘*n*-itemset’) is composed of at least two parent (*n*−1)-itemsets that partially overlap and (ii) an *n*-itemset is frequent only if its two parents are themselves frequent. Based on these ideas, an algorithm called APRIORI was developed. The basic principle of APRIORI is quite simple: at iteration *n*, the candidate *n*-itemsets are built from the frequent (*n*−1)-itemsets and are compared to a threshold λ in order to evaluate if they are frequent. If so, they are called λ-frequent itemsets. Finally, for each discrete value of λ, λ-frequent itemsets containing a maximum number of items can also be extracted by the algorithm. In our case, these maximal-frequent itemsets correspond to the SCAS to be extracted from co-occurrence matrix *B*, itself constructed from the detection of mono-IIS. At this level, Monte Carlo simulations and statistical tests can be performed to evaluate the significance of extracted SCAS (‘organized spatial distribution of mono-IIS’ versus ‘random spatial distribution of mono-IIS’).

#### (iii) Results and discussion

In this section, we report the main findings obtained from the application of the method in 15 patients with intractable partial epilepsy of temporal origin. From simultaneous intracerebral recording of mesial (hippocampus, amygdala, entorhinal cortex, temporo-basal cortex, internal temporal pole) and lateral (superior, middle and inferior temporal gyri, insulae, external temporal pole (eTP)) structures, the first objective was to characterize the subsets of structures that co-activate during the generation of interictal spikes. The second objective was to relate these subsets to particular anatomo-functional systems in the temporal lobe.

**Figure 3:**
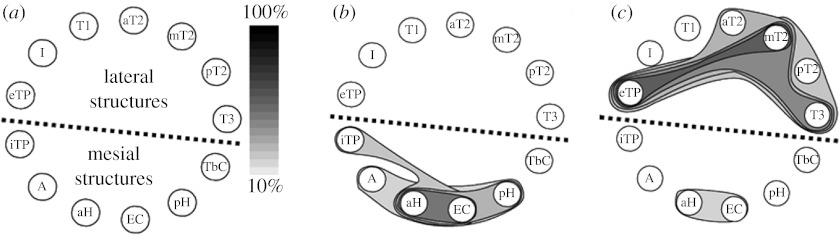
Examples of results obtained from the method illustrated in figure 2. (*a*) Recorded brain structures are placed over a circle for graphical representation. The method was applied in 15 patients with mesial TLE. Results showed that in a first subgroup of patients (8 out of 15), the networks generating epileptic spikes remain limited to the mesial structures of the temporal lobe. In the second subgroup (7 out of 15), the networks generating epileptic spikes extend far beyond the mesial structures as most lateral neocortical structures can also be involved in extracted subsets. (*b*,*c*) Examples of automatically identified SCAS in both cases. Abbreviations: A, amygdala; aH, anterior hippocampus; EC, entorhinal cortex; pH, posterior hippocampus; TbC, temporo-basal cortex; iTP, internal temporal pole; T3, middle part of inferior temporal gyrus; pT2, posterior part of middle temporal gyrus; mT2, middle part of middle temporal gyrus; aT2, anterior part of middle temporal gyrus; T1, superior temporal gyrus; I, insula; eTP, external temporal pole.

In order to give an idea, for a 1-hour interictal recording selected in each of the 15 patients, the number of multi-IIS was found to vary from 492 to 7600 (mean±s.d. 3322±2190) and 57 SCAS were automatically extracted for all patients. Figure 3 illustrates the type of result generated by the method. In this figure, the SCAS are represented in the same schematic way: cerebral structures are positioned along a circle (bottom: mesial temporal lobe, top: lateral temporal lobe) and extracted SCAS are represented using closed contour lines. Surfaces delineated by contours are coloured using a grey scale that indicates the SCAS occurrence frequency (figure 3*a*). Results obtained from the visual inspection of SCAS in the 15 cases showed that patients may be divided into two groups depending on the involvement of lateral structures of the temporal lobe. In the first subgroup of patients (8 out of 15) the networks generating epileptic spikes remain limited to the mesial structures of the temporal lobe (figure 3*b*). Therefore, a good spatial correspondence with the EZ (defined as the site of primary organization of the ictal discharge) was observed. Conversely, in a second subgroup patients (7 out of 15), the networks generating epileptic spikes extend far beyond the mesial structures as most lateral neocortical structures were found to be involved in extracted SCAS (figure 3*c*). Moreover, in all patients, at least one SCAS was localized in the mesial part of the temporal lobe with a significant incidence of the subset formed by the anterior hippocampus and entorhinal cortex. This result is consistent with respect to available data about hippocampal pathways (axons of the perforant path—major input to the hippocampus—arise principally in layers II and III of the entorhinal cortex, which is, in return, an output from the hippocampus). Among mesial structures, the temporobasal cortex and the internal part of the temporal pole were found to belong to SCAS identified in the mesial temporal lobe as well as SCAS identified in the lateral temporal lobe. This ‘pivotal role’ can mainly be interpreted by the anatomy of the temporal region as these structures are connected to both the limbic system and the lateral neocortex.

To end with, the whole method provides spatial information about mesial and/or lateral structures that are more likely to be conjointly involved in the generation of interictal spikes and allows for categorization of patients with respect to this information. This may be the first step towards better understanding of the relationship that may exist between networks involved in interictal activity and networks involved in seizure activity and therefore towards enhanced use of interictal activity for diagnosis.

### (b) Identification of subsets of structures involved in ictal activity

#### (i) Research context and problem statement

In human partial epilepsies, the identification of neuronal networks that are involved in the genesis and in the propagation of seizures is the main issue. Indeed, accurate localization and determination of these epileptogenic networks (that may extend over distant brain structures) is the prerequisite for defining the subsequent therapeutic strategy, precisely aimed at suppressing seizures by the annihilation of epileptogenic networks. The question of identifying such networks in the brain is closely related to the question of characterizing ‘abnormal’ functional couplings among neuronal ensembles possibly distributed over distant areas. Tackled questions relate to the characterization of interconnections between structures during the transition from pre-ictal activity to ictal activity: are some structures functionally connected? Can abnormal couplings be identified? How do couplings evolve during the interictal to ictal transition and during the time course of seizures? Do some structures play a leading role in the seizure generation process?

Such questions can be approached using signal processing techniques applied on electrophysiological signals recorded from these ensembles. As far as depth-EEG signals (that are local field potentials as recorded by macroscopic intracerebral electrodes) are concerned, numerous studies have been dedicated to the development and/or to the use of methods aimed at quantifying the functional coupling between recorded sites.

Formally, the two questions that are addressed can be stated as follows. Given two field signals *X*(*t*) and *Y*(*t*), respectively, recorded from two groups of neurons *G*_*X*_ and *G*_*Y*_, (i) how can we quantify, from *X*(*t*) and *Y*(*t*), the functional coupling (in the wide sense) between *G*_*X*_ and *G*_*Y*_? And (ii) how can we estimate, by signal processing, which of the four following situations is the most likely to occur: *G*_*X*_ is leading *G*_*Y*_; *G*_*Y*_ leading *G*_*X*_; *G*_*X*_ and *G*_*Y*_ mutually influence each other; and finally *G*_*X*_ and *G*_*Y*_ are independent?

To solve these issues, proposed methods all rely on the same main assumption: the functional coupling between two neuronal ensembles can be quantified by measuring the statistical relationship between the two signals, each signal arising from one neuronal ensemble. In the literature, various terms are used to denote this statistical relationship, such as the ‘degree of coupling’, the ‘degree of association’, the ‘synchronization’ or the ‘interdependency’ between signals, among others. Proposed methods can be divided into two categories depending on the assumptions regarding the relationship between signals. Linear methods include the linear cross-correlation or the coherence function. They were proposed and used to study functional couplings between brain regions during cognitive tasks or during epileptic seizures. In this field, pioneer works were initiated by Brazier (1972), who made use of the coherence function to study the propagation of epileptic activity from intracerebral recordings. They were followed by Gotman (1987), who studied interhemispheric relationships in partial seizures and by Duckrow & Spencer (1992) and Franaszczuk & Bergey (1999), who analysed synchronization mechanisms occurring at the onset of seizures. Besides these linear methods, the potential usefulness of nonlinear techniques in the field of EEG was also studied from the early 1980s. A first family of methods based on mutual information (Mars & Lopes Da Silva 1983) or on nonlinear regression (Pijn & Lopes Da Silva 1993; Wendling *et al*. 2001) was first introduced. A second family then developed based on methods coming from nonlinear physics (nonlinear dynamical systems) and chaos theory (Iasemidis *et al*. 1990; Lehnertz 1999).

#### (ii) Method: nonlinear regression analysis

Nonlinear regression analysis is a non-parametric method aimed at evaluating the dependency of a random process (a time-series signal *Y* recorded from *G*_*Y*_, for instance) on another process (signal *X* recorded from *G*_*X*_, for instance) from samples only (no data model) and independently of the type of relationship between the two processes. This method was first used in the field of EEG analysis by Pijn and colleagues (Pijn 1990; Pijn *et al*. 1992), who showed that it performed better than methods based on the linear regression or mutual information for analysing the interdependences between intracerebral EEG signals (experimental model of generalized epilepsy). An evaluation of this method was then performed based on realistic simulation of EEG signals generated by coupled populations of neurons (Wendling *et al*. 2000). We showed that this method can be applied to human intracerebral EEG data for characterizing seizure patterns (Wendling *et al*. 2001). In particular, we used it to study ictal processes in patients with TLE. Results led to the proposition of a new classification of seizures based on the early involvement of medial and/or lateral structures of the temporal lobe (Bartolomei *et al*. 2001).

Nonlinear regression analysis is a bivariate method that measures the degree of association between two variables. This measure is often referred to as the ‘nonlinear correlation coefficient *h*^2^, by analogy with the well-known linear correlation coefficient *r*^2^. Formally, the nonlinear correlation coefficient *h*^2^ is computed from the signals *X*(*t*) and *Y*(*t*), by considering that the amplitude *y* of signal *Y*(*t*+*τ*) is a perturbed function of the amplitude *x* of signal *X*(*t*) (i.e. equal to the conditional mean of *Y*(*t*+*τ*) given *X*(*t*)=*x*). The variance of the perturbation corresponds to the conditional variance of *Y*(*t*+*τ*) (i.e. the residual variance on *Y*(*t*+*τ*) after prediction of *y* values from *x* values). In practice, this conditional variance can be estimated from a piecewise linear regression curve Y(t+τ)=h(X(t)).

(3.1) $$h^{2}(\tau )=1-\frac{\text{VAR}[Y(t+\tau )/X(t)]}{\text{VAR}[Y(t+\tau )]},$$

where

(3.2) $$\text{VAR}[Y(t+\tau )/X(t)]=\hat{=}\text{arg}\;\underset{h}{\text{min}}E{\left[Y(t+\tau )-h(X(t))\right]}^{2}.$$

The computation of *h*^2^(*τ*) is reiterated for different values of the time shift *τ* between *X*(*t*) and *Y*(*t*+*τ*), leading to the time shift τ^*^, or time delay, for which *h*^2^(*τ*) is maximum:

(3.3) $$h^{2}=\underset{\tau_{\text{min}}<\tau <\tau_{\text{max}}}{\text{max}}[h^{2}(\tau )]=h^{2}(\tau^{*}),$$

*h*^2^ is the nonlinear correlation coefficient. Its values are between 0 (*X* and *Y* are independent) and 1 (*Y* is determined by *X*). The main advantage of nonlinear regression is that it does not require any assumption of the nature (linear or nonlinear) of the relationship between the two signals, bringing a solution to a common pitfall of linear regression. Another interesting property of the nonlinear correlation coefficient *h*^2^ is that it is asymmetrical: the *h*^2^ value, when computed from signal *X* to signal *Y*, differs from the value computed from signal *Y* to signal *X*. This information was shown to be useful for characterizing the causality between signals (Arnhold *et al*. 1999). Following this idea, a ‘direction index’ (named *D*) was proposed by Wendling *et al*. (2001). It makes use of both this asymmetry information and the time delay information to provide an indication about which of the two signals is most likely driving the other one. More recently, rigorous studies have confirmed the usefulness of the *h*^2^ parameter for quantifying statistical relationships between random signals (Kalitzin *et al*. 2007).

#### (iii) Results

**Figure 4:**
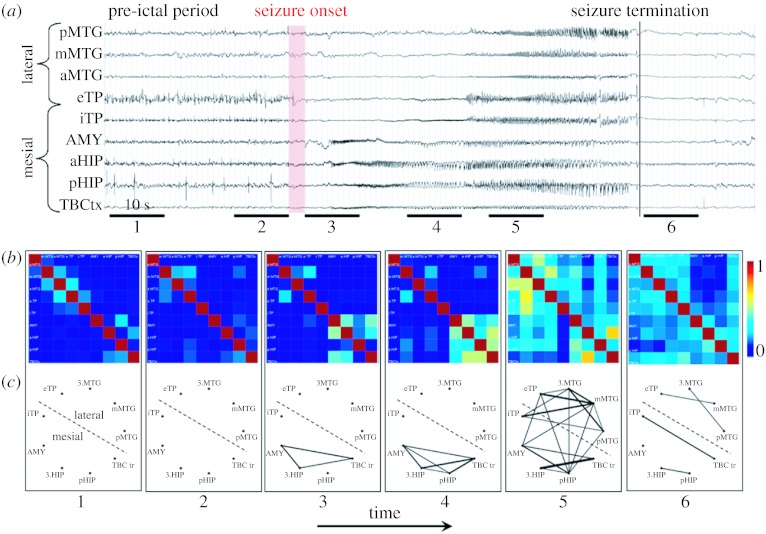
Characterization of epileptogenic networks in the temporal lobe during the transition from pre-ictal to seizure activity. (*a*) Intracerebral EEG recoding performed in a patient with mesial TLE. (*b*) Colour-coded nonlinear correlation matrices obtained from the pairwise computation of nonlinear correlation coefficient *h*^2^ over six different 10-s intervals chosen during the pre-ictal period (1, 2), the ictal period (3, 4, 5) and after seizure termination (6). (*c*) Graphical representation in which the lines indicate ‘abnormally strong’ couplings between the two considered structures (graph nodes). Only significantly high interdependencies are represented (i.e. *h*^2^ values greater than 0.32. This value corresponds to the average *h*^2^ value computed over the interictal period +2 standard deviations). Line thickness is proportional to *h*^2^ values.

An example of results obtained with nonlinear regression analysis in TLE is illustrated in figure 4. Analysed EEG signals (figure 4*a*) were recorded from intracerebral electrodes positioned in medial (amygdala, hippocampus, temporo-basal cortex, internal part of the temporal pole) and in lateral (eTP, middle temporal gyrus from anterior to posterior part) during the transition from pre-ictal activity to seizure activity. *h*^2^ values were computed pairwise (81 possible pairs for the nine signals recorded from nine structures in the temporal lobe) and averaged over 6 periods of 10 s each (two pre-ictal, three per-ictal and one post-ictal). They were then represented in two complementary ways: colour-coded nonlinear correlation matrices (figure 4*b*) and graphs (figure 4*c*) in which the nodes correspond to brains structures and in which the links are proportional to *h*^2^ values (i.e. a thick line denotes high *h*^2^ and is interpreted as strong coupling between considered structures). In nonlinear correlation matrices, all information is represented. In the graphs, only significantly high *h*^2^ values (i.e. greater than average *h*^2^ values computed over the interictal period +2 standard deviations) are displayed. One can see that dramatic modifications of *h*^2^ values (interpreted as couplings between structures) occur during the transition from ‘normal’ background activity to ictal activity. In particular, a strong increase of *h*^2^ values is observed just after the onset of the seizure marked by the appearance of fast oscillations in the limbic system (amygdala, hippocampus, temporo-basal cortex) (figure 4*b*, period 3). As the seizure develops (figure 4*b*, periods 4 and 5), the spread of ictal activity is characterized by *h*^2^ values that become higher, not only among signals from the limbic system but also between signals from the limbic system and signals from lateral neocortical structures. Detailed inspection of correlation matrices also reveals the possible strong asymmetry of the nonlinear correlation coefficient. For instance, the *h*^2^ value computed from eTP to posterior middle temporal gyrus (pMTG) over period 5 is higher than that computed in the opposite way. This denotes the possible propagation of the seizure from the eTP to the pMTG, which is plausible from the anatomo-functional point of view.

#### (iv) Discussion

This example shows the usefulness of methods aimed at characterizing the interactions between structures before and during seizures. However, the nonlinear regression analysis is not the only method that can provide such information and the user is often confronted to the delicate situation where several methods can be used and where discrepancies can be observed in the results provided by these methods. Recently, two important issues have been debated in this context: (i) whether or not nonlinear methods perform better than linear ones and (ii) whether or not frequency-dependent methods should be preferred, as some epileptic phenomena may occur in a restricted frequency domain. Regarding this second point, one should note that the frequency of EEG signals has long been considered as a key parameter that directly relates to the oscillatory behaviour of recorded brain systems. As mentioned, nonlinear methods have the capability to account for the nonlinearity of relationship. However, the fact that most of the existing ones are generally independent from frequency or at most related to large frequency sub-bands can be considered as a limitation. Conversely, linear methods cannot provide information about the possibly nonlinear nature of the relationship, but they can easily account for the signal frequency with a good resolution. The best example is probably the coherence function defined as the cross-spectral density normalized by the power spectral densities of the two signals. Indeed, linear coherence-based methods have been widely used in the field of EEG analysis. However, as underlined in Zaveri *et al*. (1999), the coherence function is estimated, in practice, from the fast Fourier transform (periodogram method) and proposed estimators are generally characterized by strong bias and variance which make the interpretation of results difficult, especially when the correlation between signals is weak. This problem was addressed by defining frequency bands. For instance, classical delta, theta, alpha, beta and gamma EEG bands were used to average the coherence function (Razoumnikova 2000) or to filter signals before computation of the cross-correlation (Nikolaev *et al*. 2001; Wendling *et al*. 2003). However, this is again not entirely satisfactory as the choice of frequency bands is critical (some important phenomena may be neglected if they are bridging two user-defined sub-bands). Some of these difficulties are addressed in Ansari-Asl *et al*. (2005). The authors proposed a novel estimator for characterizing the evolution of a linear relationship between the two signals in the time–frequency domain. This estimator, denoted by *r*^2^(*t*, *f*), is based on the computation of the Pearson product–moment correlation between EEG signals filtered in narrow and overlapping frequency bands (a continuous filter bank is used). Briefly, this estimator has two advantages: (i) no assumption on frequency bands is required and (ii) although it asymptotically behaves such as the classical coherence estimator, it was shown to perform better in terms of bias and variance under certain conditions about frequency-dependent time delay values between signals.

**Figure 5:**
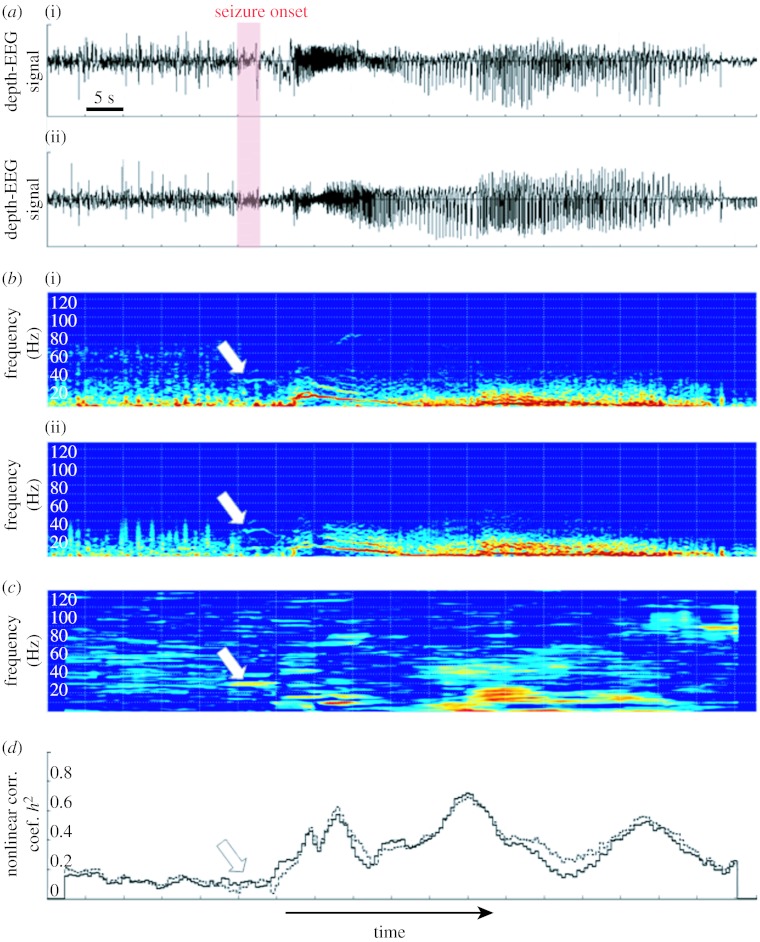
Results obtained on real data. (*a*) Depth-EEG signals recorded from (i) amygdala (AMY) and (ii) hippocampus (HIP) in a human during transition to seizure activity in temporal lobe epilepsy. (*b*(i)(ii)) Spectrograms corresponding to depth-EEG signals obtained from short-term Fourier transform. (*c*) Estimated relationship between the two signals in the time–frequency plane using the *r*^2^(*t*,*f*) method (frequency dependent). The method reveals a synchronization process well localized in frequency at seizure onset. (*d*) Estimated relationship between the two signals using the *h*^2^(*t*) nonlinear regression analysis method (frequency independent). The method is not sensitive to the narrow-band synchronization process at seizure onset. Solid curve, AMY→a HIP; dotted curve, a HIP→AMY.

An example is provided in figure 5, which shows two depth-EEG signals recorded from the amygdala and the anterior hippocampus during the transition to seizure in a patient with TLE (figure 5*a*). Both signals are non-stationary as depicted in figure 5*b*, which provides respective spectrograms. Typically, at seizure onset, a high-frequency activity (fast oscillations approx. 30 Hz) is observed in the brain structures involved in the seizure process (arrows on the spectrograms). The time–frequency characterization of the relationship between the two signals using the *r*^2^(*t*, *f*) estimator is illustrated in figure 5*c*. It reveals that a strong relationship exists between the narrow-band activities generated by the two structures and previously observed on spectrograms (approx. 30 Hz, see arrow). Such ‘hypersynchronization’ would be very difficult to detect visually. Moreover, this phenomenon is not revealed by the frequency-independent nonlinear regression analysis method, as illustrated in figure 5*d*. Indeed, any method that globally performs an averaging over the frequency domain can be ‘blind’ to synchronization processes highly localized in frequency. Therefore, this example shows the usefulness of frequency-dependent methods able to track the evolution of the relationship between signals in the time–frequency plane with a good resolution.

## 4. Conclusions

Among electrophysiological investigation methods, EEG still occupies an important place as it allows for studying brain activity in general, and epileptic processes in particular, with appropriate time resolution.

Epilepsy is a complex dynamical disease (Lopes da Silva *et al*. 2003). The term ‘epilepsy’ refers to a wide variety of neurological syndromes and disorders. In this panorama, partial epilepsies in which seizures start in a relatively circumscribed area of the brain represent 60 per cent of the cases. In this paper, we focused on pharmacoresistant partial epilepsies that are considered as severe forms of epilepsy (since significant reduction of the frequency of seizures cannot be obtained with drugs) and in which surgery may be indicated.

Scalp-EEG or depth-EEG signals recorded in patients with drug-resistant partial epilepsy convey important information about epileptogenic networks that must be localized and understood prior to any therapeutic procedure. However, this information, often subtle, is ‘hidden’ in the signals. It is precisely the role of signal processing to reveal this information and put it into a ‘coherent and interpretable picture’ that can participate in the elaboration of the decision about which part of the network should be operated on in order to suppress seizures (i.e. the therapeutic strategy).

The methods presented in this paper apply to intracerebral EEG. We think that progress must still be accomplished in the analysis of scalp EEG recorded in patients with partial epilepsy. This modality has the enormous advantage of being non-invasive. However, it only allows for a global recording of the brain activity, conversely to intracerebral EEG in which electrodes implanted in brain structures record local field potentials. Therefore, the problem of identifying epileptogenic networks from scalp recordings is highly complex in a context where signals are also largely contaminated by patient-related artefacts (such as muscular activity due to the movements of the patient during seizures). Among recent advances in quantified scalp EEG analysis, one can mention the use of blind source separation techniques for the removal of ocular artefacts and noise (see James & Hesse (2005) for a review on the use of independent component analysis for biomedical signals and Kachenoura *et al*. (2008) for performance comparisons of ICA methods or the use of nonlinear regression for the lateralization of seizures in TLE (Caparos *et al*. 2006)).

Finally, presented and quoted methods belong to a more general approach, often referred to as data-driven processing. They provide quantities that participate in the description of the observations and subsequently in the decision that can be taken from EEG signals. We think that such a descriptive approach can be valuably complemented by model-driven processing aimed at providing clues about the pathophysiological mechanisms involved into the generation of signals. In the field of epilepsy, several studies have already shown that neurophysiologically relevant computational models can be used to interpret quantities provided by signal processing methods. For instance, it was shown that models of coupled populations of neurons can be used to explain the time course of interdependencies between depth-EEG signals during partial seizures (Wendling *et al*. 2001). Following the same idea, the statistics of occurrence of absence seizures could be explained by the bistable property of a model of the thalamo-cortical loop (Suffczynski *et al*. 2005). More generally, the development of computational models is rapidly growing as there is also a need for integrating and structuring the tremendous amount of data that are continually accumulating in epilepsy research at both clinical and experimental levels (Suffczynski *et al*. 2006; Chakravarthy *et al*. 2007).
